# AtTheViewBox: Scrolling Past PowerPoints to a Novel Web-Based Solution for Interactive Case-Based Presentations

**DOI:** 10.1007/s10278-025-01620-5

**Published:** 2025-09-16

**Authors:** Michael Fei, Dane Van Tassel, Ianto Xi, Vineeth Gangaram

**Affiliations:** 1 Department of Radiology, Creighton University School of Medicine, Phoenix, AZ USA; 2 Department of Radiology, Hospital of the University of Pennsylvania, Philadelphia, PA USA

**Keywords:** Medical education, Case-based learning, Web-based application, Virtual learning

## Abstract

Traditional radiology education relies heavily on PowerPoint presentations with static 2D images, which fail to replicate the interactive nature of reading radiological studies at a workstation. There is a growing need for an interactive, case-based platform that enables real-time collaboration in presentations. This study introduces AtTheViewBox, a web-based application designed to integrate DICOM images into presentations, offering a more dynamic and interactive learning experience. AtTheViewBox was developed using open-source libraries, including React, CornerstoneJS, and Supabase. The application allows users to embed DICOM images in slide presentations via iframes, enabling standard functionalities at a radiology workstation like scrolling, zooming, and windowing. A survey was conducted among radiology residents and educators from four academic institutions to assess the utility and ease of use of AtTheViewBox compared to traditional teaching methods. Among 27 radiology residents surveyed, 100% agreed that AtTheViewBox would enhance their case-based learning experience, with 93% preferring it over static images or videos. Among 30 educators, the application received an average usefulness rating of 9.5/10. Additionally, 63% of educators found AtTheViewBox as easy or easier to use than their current methods. AtTheViewBox effectively modernizes radiology education by enabling interactive DICOM integration in presentations. This tool enhances learning by mimicking workstation experiences and fostering real-time collaboration. The overwhelmingly positive reception suggests that AtTheViewBox addresses key limitations in current teaching methodologies and has the potential to become a standard in radiology education.

**Supplementary Information:**

The online version contains supplementary material available at 10.1007/s10278-025-01620-5.

## Introduction

Just as clinical practice has evolved over the past decades to reflect changes in available diagnostic and therapeutic technology, so too must educational practice. Throughout the past 20 years, active case-based learning has proven to be a more effective way to learn, hence the addition of case-based lectures in medical school and resident education [1]. In a non-radiological setting, tools have been utilized to create an interactive case-based environment. Educators today have increasingly embraced tools designed to support hybrid learning environments, leveraging a variety of interactive websites and applications [2]. For instance, educators can embed Poll Everywhere (Poll Everywhere, San Francisco, CA) questions or use Kahoot (Goldman Sachs Asset Management, New York City, NY), which allows a group of users to individually engage in questions from a singular presentation. These technologies work well for non-radiological education, but are incomplete/limited for radiology education, particularly for advanced imaging review of ultrasound, Magnetic resonance imaging (MRI), and computerized tomography (CT).

While clinical radiology has continued to increase in digital sophistication, the teaching of radiology has not kept pace, with lectures largely taught through PowerPoint (Microsoft Corp, Seattle, WA) presentations with static 2D images, typically screenshots taken from PACS, which has access to a full DICOM image [3–5]. This current lecture experience does not translate to the workflow of reading radiological studies at the workstation. This is especially true with the increasing volume of volumetric radiological studies (CT, MRI, CT) that cannot be fully represented in PowerPoint [6], which is designed for general consumer use and is not tuned for radiology data. This leads presenters to display cases as static images, GIFs, videos, or stacks of slices, which may be insufficient for highlighting certain pathologies compared to reviewing those cases at a workstation, particularly because these radiological studies are typically rich in spatial and color-depth information.

Web-based tools have been created to display Digital Imaging and Communications in Medicine (DICOM) studies and demonstrate that a workstation-level experience can be achieved. While long being the de facto tool for slide-based presentations, PowerPoint has limitations for extending functionality. Web-based presentations are becoming more popular (Canva for Radiopedia e-posters), and many web presentation software programs provide the ability to create extensions, which could be used to natively support DICOM files.

Collaborative work and learning in real time have been a large focal point in many areas of study, showing strong efficacy [7, 8]. Yet there seems to be unnecessary friction in creating a similar experience in radiology education. The closest experience is granting remote control during a video call; however, the latency caused by transmitting the video creates an experience with slow response times and a poor user experience.

This study aims to create a web-based application, AtTheViewBox, which allows.

1. Educators to easily embed cases into slide presentations in a way that allows the educator to match the experience of teaching “at the viewbox” (scrollable, zoomable, windowable DICOMs)
2. Residents and/or students to interact with the case (scroll, zoom, window, point to findings), particularly for hybrid in-person/virtual lectures.

Another goal of this study is to assess the utility and ease of use of AtTheViewBox by surveying residents to understand whether this tool enhanced their experience as learners and surveying senior residents and attendings as educators.

## Method

### Design/Features

AtTheViewBox is a web application that can be used with any web browser, allowing it to be used as a stand-alone website. Because it is a web-based application, AtTheViewBox was also built to be embedded in presentations using iframes. An iframe is a web element that allows users to load and display a website within another website. Given this, AtTheViewBox is built with this set of features:

1. Loading DICOM cases: Many presentation web-based platforms like slides.com or Canva support iframes, which allow users to paste an AtTheViewBox Uniform Resource Locator (URL) to embed it within the presentation. This allows users to easily add cases to a presentation simply by using a different URL.
2. Interact with DICOM cases: Within the iframe, users can perform the standard features at the workstation like scrolling, windowing, panning, and zooming.
3. Create and share sessions: Users can start a session room to generate a session URL and a quick-response (QR) code. Other users scan the QR with their mobile device or use the session URL to join the room. When the presenter is ready for a new case, presenters are able to switch the whole room to a new case.
4. Alter presentation type: There are two room modes, Team mode and Presentation mode. In Team mode, any user can share their interactions with everyone else in the room, while in Presentation mode, users can only share their interactions with the presenter screen. To share, the users hold down the target button on the bottom left side of the window, and their image will be mirrored to others.

**Fig. 1:**
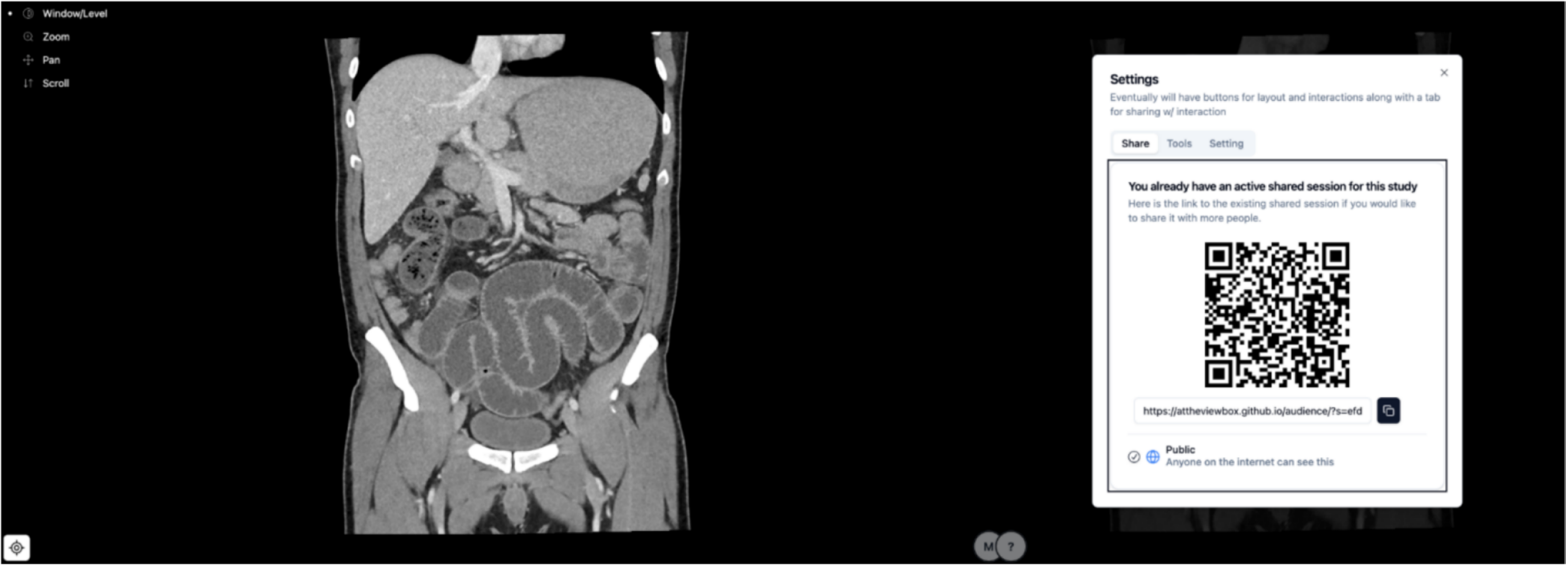
The user interface of AtTheViewBox. The DICOM tools are in the top left corner. The users in the session are in the bottom right-hand corner. Clicking the icon in the bottom left corner opens the QR code, and holding the icon allows users to share

The user interface can be viewed in Fig. 1, and a demonstration can be viewed in Video 1. Refer to https://mfei1225.github.io/AtTheViewBox_Demo_Site/ for a demonstration and how to get started.

### Architecture

**Fig. 2:**
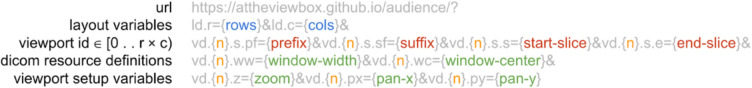
Structure of URL. Variables in blue define the radiology studies that are displayed. Variables in red define the Amazon S3 bucket location. Variables in green define the initial setting the DICOM is viewed with

**Fig. 3:**
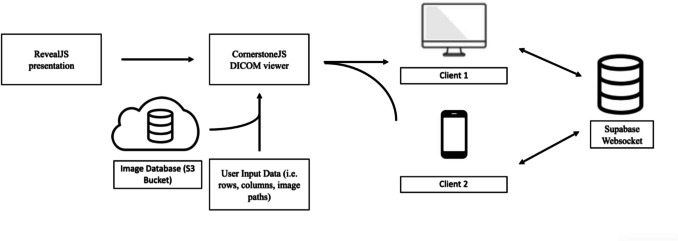
High-level design of the frontend and backend architecture of AtTheViewBox

AtTheViewBox is a web-based application built using open-source libraries and services. DICOM images are from an Amazon S3 bucket (Amazon, Seattle WA). The front end uses the React framework and CornerstoneJS to load and manage the DICOM images. All DICOM image information is stored within the URL, allowing URLs to be easily modified to specific cases and shared with others, which can be viewed in Fig. 2. The URL structure also allows multiple sequences to be included in one case for cases where multiple sequences are needed, which can be viewed in Video 1. The backend is built using Supabase, an open-source database infrastructure that manages user authentication and real-time sharing. Supabase’s Realtime application programming interface, which uses WebSockets, is used for real-time collaborative sharing [9]. WebSocket is a protocol that allows for two-way communication between different clients, allowing fast and responsive interaction to be shared with multiple users, traditionally used in applications like group messages or online games. A high level design can be viewed in Fig. 3. All code is open-source and can be viewed here https://github.com/AtTheViewbox/audience.

### Survey

A survey was conducted among radiology residents to gauge their interest in using AtTheViewBox to learn radiology from a student’s perspective. A live demonstration was created with Slide.com and AtTheViewBox for the residency’s upcoming emergency readiness workshop and sent to a group of radiology residents from three radiology academic institutes: University of Pennsylvania, Emory University, and Mallinckrodt Radiology Departments. Questions were used to gauge the utility of AtTheViewBox compared to the current standards of implementing radiological studies into the presentation.

An additional survey was conducted among attendings and residents from four academic institutes: University of Pennsylvania, Creighton University, Emory University, and Mallinckrodt Radiology Departments. from the perspective of an educator. Another demo presentation with three small bowel obstruction cases was created with Slide.com and AtTheViewBox. The survey asked about the utility and their confidence in their ability to create a similar presentation. The same educators were shown a video of the no-code interface in AtTheViewBox and had the opportunity to create their own cases. They were surveyed again on their confidence in their ability to create a similar presentation. A *t*-test was performed between the pre- and post-tutorial confidence levels to assess if AtTheViewBox was a tool educators felt comfortable using to create embedded case-based presentations. This educational study met the exclusion criteria for Institutional Review Board approval.

## Result

Unanimously, 27 of the 27 (100%) first-year through fourth-year radiology residents expressed that the demo tool would improve their experience with case-based learning. Twenty-five of 27 (93%) residents surveyed stated they would prefer embedded DICOMs over current alternatives such as static images, GIFs, and videos. Regarding case navigation, 26 of 27 (96%) residents reported they would prefer personal device syncing like what is provided by AtTheViewBox over current alternatives like a shared Bluetooth mouse or directing the lecturer to scroll.

There was a total of 30 responses from educators. In regard to educators’ perception of AtTheViewBox, on a Likert scale of 1–10 on usefulness, with 10 being “strong asset to medical education,” they rated an average of 9.5/10 on the usefulness format. An open-ended question was asked to explain the reasoning for their rating. Qualitatively, the comments aligned with the goals of AtTheViewBox, with nine comments mentioning that AtTheViewBox more closely replicated reading at the workstation, and an educator noting, “This tool successfully creates a realistic model that both allows the user to find and critically think about the pathology for themselves.” When asked on a Likert scale of 1–10 about their confidence in creating a similar presentation, with 10 being “100% chance they could create a similar presentation,” they rated an average of 5.5/10. When asked to explain their rating, many noted that there was a technical barrier to creating something like this. Even participants with the technical knowledge noted that due to their limited time, they are unlikely to create something similar. After viewing the tutorial on AtTheViewBox, the average rating of their confidence in creating a similar presentation was 8.3/10, a significant increase with a *p*-value of 1.56e − 7. Nineteen of the 30 (63%) educators also noted that the process with AtTheViewBox is equivalent to or easier than their current approach of adding cases to their presentations.

## Discussion

Traditionally, radiology was practiced and taught with attendings and residents going to the view box to read films. Over the past 30 years, the workflow at the workstation has been digitized. While educational content has been digitized, it has done so with off-the-shelf educational software like PowerPoint, which provides suboptimal support for sharing radiology cases.

Additionally, even when cases are embedded, audience interaction modes can be unwieldy, particularly for residencies with multi-site lectures. With classic PowerPoint presentations, the lecturer often presents image slices with the key findings. This greatly reduces the learning experience as a large portion of radiology consists of steps that lead up to the key findings, such as search patterns and windowing. Many skills from interpreting DICOMS are lost if students are learning how to read a 3D image from 2D images. By going through DICOMs individually, students are able to practice these techniques. Moreover, students are able to think critically about where pathologies are present, how pathologies evolve in different parts of the body, and how pathologies interact with different anatomies in the body. An interactive tool that allows the student to follow as an educator scrolls through a DICOM can be even more beneficial for medical students. In these cases, medical students are just beginning to familiarize themselves with radiologic images and may not have the expertise to extrapolate information and translate findings from one image slice to another. A similar case can be made when teaching radiology didactics to non-radiology specialties.

AtTheViewBox provides a tool that, as this study has shown, is both desired by learners and can bridge the technical gap for educators to implement. AtTheViewBox currently allows a flexible use case with its two sharing modes. The Team mode can be used in resident-attending pairs or smaller group settings, where cases would be discussed back and forth as live sharing is available to everyone in the room. The Presentation mode can be used in large lectures or presentations where the live sharing is broadcast to the presenter’s screen. This allows students to continue looking through the case even when other students are sharing their findings. This collaborative system mimics the software applications used in other areas of study, like Google Docs (Google, Mountain View, CA), where multiple users can work on the same Word document at the same time. Similar tools have been created for users to learn about computer science and programming through applications like HackerRank (HackerRank, Mountain View, CA) or Visual Studio Live Share (Microsoft Corp, Seattle, WA).

Other similar applications like Pacsbin (Orion Medical Technologies, Baltimore, MD) have been created that can be used for case-based learning. Pacsbin.com is a great tool to view de-identified DICOMs, where users can interact with the image. Some studies have used it in creating a similar case-based learning experience [10–13]. However, the overall design was built for asynchronous learning, and not optimized for a presentation and a lecture setting. Smith et al. primarily used Pacsbin to create a self-directed learning course [13]. Sugi et al. present the idea of sharing Pacsbin cases with QR codes [10]. However, once students have viewed the case, Sugi et al. do not offer an elegant solution for sharing the findings. McRoy et al. used Pacsbin and Zoom’s remote control feature to allow users to share [12]. However, this presents extra complexities, as all users have to log into a Zoom meeting regardless of an in-person lecture or presentation. Additionally, the remote control offers a slow and unresponsive user experience. AtTheViewBox simplifies and streamlines the task that many of these other papers are trying to achieve with a multitude of other tools.

One current limitation of the AtTheViewBox is that it can only display cases stored in publicly accessible DICOM files hosted on Amazon S3 bucket. As a result, institutions or individuals have to set up a means to host their own DICOM files. This barrier is largely due to the cost associated with using Amazon S3 for large-scale storage. We are currently exploring other storage options like Cloudflare R2 or IBM Cloud Object Storage, which could significantly reduce costs and ease the financial burden on users. Ultimately, getting this setup would allow users to upload cases directly to AtTheViewBox. It would also allow us to curate and maintain a permanent set of teaching files gathered through multiple institutions. Another limitation of AtTheViewBox is its use in PowerPoint presentations limited by software version compatibility, plugin approvals, and institutional permissions. While PowerPoint remains the dominant presentation tool, web-based presentation software is becoming increasingly common each year. We are actively exploring ways to bring our tools to educators on the platforms they are more familiar with, but we also anticipate a broader shift toward web-based presentations due to their ease of sharing, accessibility, and collaboration seen by the growing adoption of rPosters on Radiopaedia.

## Conclusion

As new medical findings and medical education evolve, didactic methodologies should reflect these shifts. This is especially true in an era where learning often takes place online, and many students use digital materials. A radiologist’s primary role is to interpret digital images and effectively communicate their findings, but didactics in this field have yet to catch up to the needs of the present-day learner. AtTheViewBox is an application that garners interest from both radiology educators and learners. The open-source web-based application is a step to modernize radiology education by allowing individuals to embed full DICOM cases in a presentation and see real-time changes. Ultimately, this can enrich the educational experience by replicating reading at the workstation and oral boards.

## Supplementary Information

Below is the link to the electronic supplementary material.

ESM 1 (MOV 123MB)
